# Refining associations between *TAS2R38 *diplotypes and the 6-*n*-propylthiouracil (PROP) taste test: findings from the Avon Longitudinal Study of Parents and Children

**DOI:** 10.1186/1471-2156-8-51

**Published:** 2007-07-28

**Authors:** Nicholas J Timpson, Jon Heron, Ian NM Day, Susan M Ring, Linda M Bartoshuk, Jeremy Horwood, Pauline Emmett, George Davey-Smith

**Affiliations:** 1 Department of Social Medicine, Bristol University, Bristol, UK; 2 ALSPAC, Department of Social Medicine, Bristol University, Bristol, UK; 3 University of Florida, Dept. of Community Dentistry and Behavioral Science, Gainesvile, USA; 4 The Wellcome Trust Centre for Human Genetics, Oxford University, Oxford, UK

## Abstract

**Background:**

Previous investigations have highlighted the importance of genetic variation in the determination of bitter tasting ability, however have left unaddressed questions as to within group variation in tasting ability or the possibility of genetic prescription of intermediate tasting ability. Our aim was to examine the relationships between bitter tasting ability and variation at the *TAS2R38 *locus and to assess the role of psychosocial factors in explaining residual, within group, variation in tasting ability.

**Results:**

In a large sample of children from the Avon Longitudinal Study of Parents and Children, we confirmed an association between bitter compound tasting ability and *TAS2R38 *variation and found evidence of a genetic association with intermediate tasting ability. Antisocial behaviour, social class and depression showed no consistent relationship with the distribution of taste test scores.

**Conclusion:**

Factors which could influence a child's chosen taste score, extra to taste receptor variation, appeared not to show relationships with test score. Observed spread in the distribution of the taste test scores *within *hypothesised taster groups, is likely to be, or at least in part, due to physiological differentiation regulated by other genetic contributors. Results confirm relationships between genetic variation and bitter compound tasting ability in a large sample, and suggest that *TAS2R38 *variation may also be associated with intermediate tasting ability.

## Background

Variation in the ability to taste PTC, PROP and related compounds has been recognised as one of the classical markers of population genetics. Since the seminal findings of Blakeslee and Fox [1,2], the distribution of PTC/PROP tasting has been extensively analysed. This variation has been noted to vary from the extremes of "taste blindness" (a lack of sensitivity, or non-taster of PTC/PROP), to apparent "super tasting" (an extra sensitive reaction to the bitterness of PTC/PROP) and has shown marked variation in the distribution of such traits across populations (taste blindness ranging from 3% in West Africa, to 6–23% in China, 40% in India and around 30% in North American Caucasian populations [3,4]).

Conventional assessment of bitter tasting ability has been by PTC (phenylthiocarbamide)/PROP taste challenge and response assessment. It was found that all bitter compounds containing the thiocyanate (N-C = S) moiety elicit bimodal patterns of response [5]. In addition to this it has been shown that PTC taste responses are strongly correlated with all of these compounds [6], as is the case for PROP [3].

Initial genetic analyses presented indications of bitter compound tasting ability as a complex genetic trait, but provided little specific evidence as to possible causal, or robustly associated genetic components [4,7-11]. However, specific positional cloning efforts have since identified a region of chromosome 7 (in the *TAS2R38 *gene), which has shown patterns of haplotypic association which are associated with specific measurements of bitter tasting ability [12]. This has provided direct evidence of a physiological link between genetic variation and tasting ability and has prompted hypotheses as to the possible relevance of bitter tasting for ultimate diet choice and related health [13-15]. We note that the human genome project has now identified more than twenty genes for bitter taste [16], likely to also associate with health effects.

Existing studies assessing relationships between *TAS2R38 *haplotype variation and specific bitter tasting ability have limited samples sizes and have largely been restricted to the assessment of relationships between genetic variation at the TAS2R38 locus and the binary measure "taster/non-taster". Whilst there is evidence as to the existence of this association, there has not to date, been large-scale replication or refinement of these observations. Furthermore, whilst diplotypes of the *TAS2R38 *gene locus are thought to prescribe one's ability to detect the bitter compounds PROP and PTC (PROP representing a suboptimal, but effective ligand for the TAS2R38 receptor), little its known about the effects/association of haplotypes at intermediate/lower frequency or the cause of the distribution of bitter tasting ability within haplotype defined groups.

Studies into the psychophysiology of PROP detection have suggested that in addition to tasters and non-tasters, there also "supertasters" of bitter compounds. Supertasters of PROP are believed to be tasters who also have unusual tongue anatomy: a high density of fungiform papillae, the structures that house taste buds. Supertasting is not linked to variation et the *TAS2R38 *locus [15].

The present analyses are focussed on "taster" or "non-taster" as the only previous grouping of note related to *TAS2R38 *locus variation. It is therefore not the existence of super tasting that is in question, more the refinement of the currently crude relationships recorded between genetic variation at the *TAS2R38 *locus and the ability to taste specific bitter compounds. It is predicted, that with a study of this magnitude, the observation of the rare AA haplotype at the *TAS2R38 *locus within the ALSPAC cohort will allow investigation of its potential link to intermediate tasting ability [17].

Such an investigation, together with the suggestion of subjectivity in the rating of PROP taste test scores [18], also raises the question as to the determination of the distribution of PROP taste test scores within genetically prescribed tasting groups. Whilst physiological factors such as the number of taste buds may be instrumental in the determination of this variation [19], it has been suggested that psychological/behavioural factors play some role [20].

Here we analyse PROP bitter taste test scores collected in the ALSPAC child cohort and their relation to known variation at the *TAS2R38 *locus. Also, the availability of a series of psychological/behavioural measures reflecting factors thought to influence the ultimate response of a child to this taste challenge, has allowed the investigation of residual variation in bitter tasting ability. As such, we aim to comment on the possible origins of the distribution of taste scores found within the prior defined haplotypic taste groups.

## Results

Of the 13988 cases/pregnancies in the ALSPAC cohort, samples from n = 9765 children and n = 8736 mothers formed a working population of n = 12234 unrelated individuals (all genotyped children and unrelated mothers where children were not available, comprising 83% of the cohort) available for haplotypic reconstruction. Of this group, n = 8 had one missing genotype and n = 174 had missing data for two genotypes. Genotyping of the *TAS2R38 *variants P49A and A262V yielded minor allele frequencies of 0.40 and 0.45 respectively, Table 2. From these genotyping results, haplotypic reconstruction resolved the common haplotypes AV and PA at frequencies of 0.55 (SE 0.0004) and 0.40(SE 0.0004) within this population, Table 3. The rarer haplotypes AA and PV were observed at frequencies 0.05 (SE 0.0003) and 0.001(SE 0.00008).

**Table 2:** Allele frequencies at variant loci *TAS2R38 *P49A and A262V in the ALSPAC sample

Nucleotide	Amino Acid	Allele	Protein code	Frequency in current study	p*	Comparative frequencies (Kim et al 2003)
145	49	G	Ala	0.60 (n = 10977)	0.9	0.64
		C	Pro	0.40 (n = 7467)		0.36
262	262	T	Val	0.55 (n = 10147)	0.7	0.62
		C	Ala	0.45 (n = 8405)		0.38

* indicates p value for test of Hardy Weinberg Equilibrium (exact test [37])

**Table 3:** Haplotype frequencies at variant loci *TAS2R38 *P49A and A262V in the ALSPAC sample

Haplotype	P49A	A262V	Observed Frequency	Comparative frequencies (Kim et al 2003)
AV	G	T	0.546	0.47
AA	G	C	0.047	-
PA	C	C	0.406	0.49

**Figure 1:**
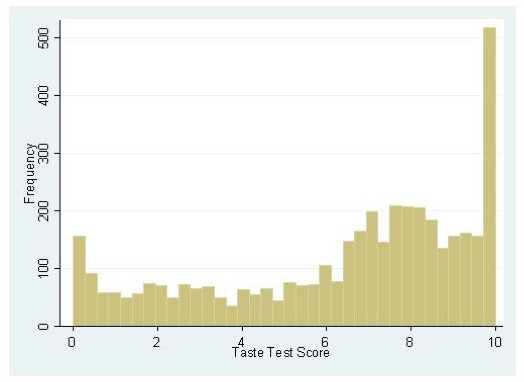
**Distribution of PROP taste scores in the ALSPAC cohort by frequency**. Taste test score derived from a general labelled magnitude scale.

**Figure 2:**
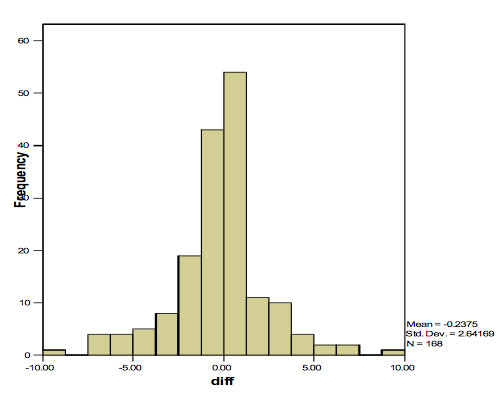
**Distribution of differences between multiple taste test assessments in the ALSPAC cohort**. Distribution of differences observed between general visual analogue scale scores for the description PROP taste test results within 168 repeat tested children from the ALSPAC cohort. Mean difference between two measures is 0.24(0.16, 0.64), p = 0.25.

Haplotype frequencies (common haplotypes representing ~99% of all variation) are derived from a sample base n = 12234 (derived from n = 9399 children, n = 2661 mothers where child has no genotype data and 174 missing data points). Haplotypes for P49A/A262V genotypes obey Hardy Weinberg Equilibrium (p = 0.8, chitesti [38]). The rare haplotype PV was observed in the ALSPAC population at a frequency of 0.001.

n = 4795 children completed a PROP taste test analysis and were carried forward into further analysis. Figure 1 shows the distribution of PROP taste test scores in the ALSPAC population. From the 168 children who completed the PROP taste test twice, the correlation of scores between both tests was 0.62 (r^2 ^= 0.39). This relationship yielded a regression coefficient of 0.61 (0.49, 0.72). p =< 0.001. Figure 2 shows the distribution of differences that exist between taste test scores for the 168 children over these two time points. The mean time between the two measurements for this group was 33.4 days.

The combination of PROP taste test data with haplotypic information yielded a test population of n = 4178 children. Of note, in analysis of PROP taste test scores by PC haplotype, none of the reconstructed haplotypic values fell below the cut off posterior reconstruction threshold probability of 0.8.

The distribution of PROP taste test scores by haplotypic combination (diplotype) is shown in Table 4. PROP taste test score was observed to be lowest for the haplotypically predicted non-tasters (AV/AV – median 3.72, IQR [5.2]), whilst predicted tasters had high PROP taste test scores with the highest being for homozygous taster haplotypes (PA/PA – median 8.07, IQR [2.5]). Of note, whilst the carriage of the PA haplotype appeared to confer tasting ability throughout, the presence of the rarer heterozygote AV/AA showed evidence for intermediate tasting ability.

**Table 4:** Median PROP taste test scores (on a scale of 0–10 with 10 being the most intense taste response) by diplotype in the ALSPAC cohort

Diplotype	N	Predicted PROP tasting ability	Median PROP Taste Score	IQR
AV/AV	1202	Non-taster	3.72	5.2
AV/AA	202	(Intermediate)	5.37	5
AV/PA	1893	Taster	7.48	2.6
AA/PA	140	Taster	7.58	2.85
PA/PA	727	Taster	8.07	2.5

Common haplotypic combinations shown above account for >99% of the sample population included in analysis. IQR represents interquartile range. Derived from a sample base of 4178 (i.e. all those remaining with PROP taste test scores post haplotypic exclusion).

Pairwise differences in mean PROP taste test score showed expected patterns according to diplotype comparison (Table 5). The largest difference in tasting response was seen between diplotypes AV/AV (non-taster) and PA/PA (homozygote taster), (4.35 95% CI [4.13, 4.56]). In contrast, the smallest difference between diplotypes AV/PA (heterozygote taster) and AA/PA (heterozygote taster, (0.10 95% CI [-0.26, 0.46]). The presence of the rarer haplotype AA (when in comparison to either taster or non-taster diplotypes) exhibited moderate pairwise differences (e.g. AV/AA versus AV/AV pairwise difference of 1.65 95% CI [1.21, 2.10] and AV/AA versus PA/PA pairwise difference of 2.70, 95% CI [2.26, 3.13]).

**Table 5:** Pairwise differences of mean PROP taste test score by diplotype in ALSPAC

*	**AV/AV**	**AV/AA**	**AV/PA**	**AA/PA**	**PA/PA**
**AV/AV**	*	Non-taster Intermediate	Non-taster Taster (Het)	Non-taster Taster (Het)	Non-taster Taster (Hom)
**AV/AA**	1.65 (1.21, 2.10)	*	Intermediate Taster (Het)	Intermediate Taster (Het)	Intermediate Taster (Hom)
**AV/PA**	3.76 (3.57, 3.95)	2.11 (1.68, 2.53)	*	Taster (Het) Taster (Het)	Taster (Het) Taster (Hom)
**AA/PA**	3.86 (3.48, 4.24)	2.10 (1.67, 2.75)	0.10 (-0.26, 0.46)	*	Taster (Het) Taster (Hom)
**PA/PA**	4.35 (4.13, 4.56)	2.70 (2.26, 3.13)	0.59 (0.42, 0.76)	0.49 (0.11, 0.86)	*

Pairwise difference of mean PROP taste score displayed with predicted tasting status in opposing matrix space. Estimates and 95% CI derived from categorical regression of taste test score by diplotype with robust SE.

**Figure 3:**
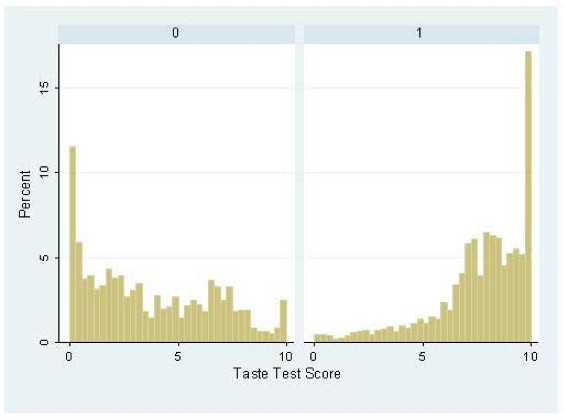
**Distribution of PROP taste test scores by genetic prediction of tasting ability in the ALSPAC cohort**. Test groups here correspond to 0 = non-tasters as defined by the homozygous carriage of the TAS2R38 haplotype AV; 1 = tasters as defined by the homozygous or heterozygous carriage of the TAS2R38 haplotype PA. Ranksum analysis for comparison of random points from these two distributions yields p =< 0.001.

**Figure 4:**
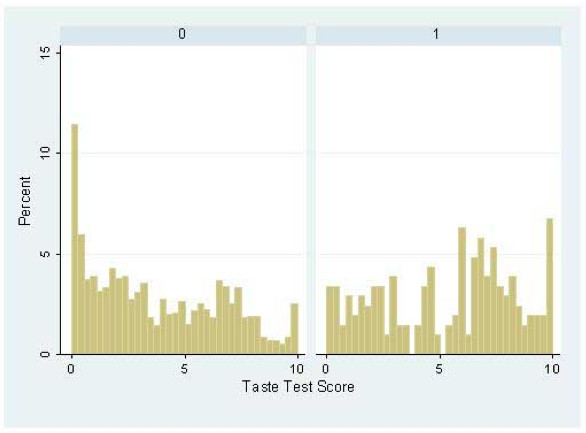
**Disrtibution of PROP taste test scores by the rare AA haplotype in the ALSPAC cohort**. Test groups here correspond to 1 = any carriage of the rare AA haplotype, excluding carriers of the common PA taster haplotype, i.e. predicted intermediate tasters; 0 = all other individuals. Ranksum analysis for comparison of random points from these two distributions yields p =< 0.001.

From these observations, the generation of predicted tasting ability groups was permitted and allowed the analysis of the distribution taste scores between them, Figure 3. Clear differences in the distribution of PROP taste test scores were observed between the haplotypically prescribed tasting groups, as confirmed by a rank sum test for differences between these groups (z = -35.302, p =< 0.001). Furthermore, the creation of a third group defined by the carriage of the rare haplotype AA (Figure 4), whilst only observed in relatively small numbers (n = 207 excluding those with the common PA taster haplotype on the basis of apparently dominant effects) revealed patterns indicative of intermediate tasting ability when compared to the main group distributions, this was again confirmed by a rank sum test for differences between these groups (z = -7.132, p =< 0.001) (Figure 2).

Analysis of quartiles of taste test score with respect to their relationship with factors which could potentially explain the spread of PROP taste test results within the haplotype defined test groupings seen in Figure 3., showed no strong evidence of trend effect. When assessed in both homozygote taster predicted individuals and homozygote non-taster predicted individuals (in efforts to avoid noise from differing genetic background), social class, depression and antisocial behaviour (thought to be potentially influencing child's reporting of taste sensation despite physiological response) appeared to show no consistent relationship with the spread of taste test results, tables 6 and 7.

**Table 6:** Behavioural variables (number (%)) by quartile of PROP taste score for AV/AV homozygotes (non-tasters).

Homozygote non-taster (AV/AV)	Manual social class (n = 1055)	Antisocial activities (n = 1190)	Depressed tendency (n = 1174)
1	129	55	83
	(48.86)	(18.46)	(28.04)
2	130	26	75
	(47.79)	(8.61)	(25.34)
3	119	49	68
	(45.25)	(16.23)	(23.05)
4	116	42	88
	(45.31)	(14.58)	(30.66)
linear test, p	0.3	0.7	0.01

Note, reference groups are not shown. 1, 2, 3, 4 represent grouping by quartiles of PROP taste test (general labelled magnitude scale).

**Table 7:** Behavioural variables (number (%)) by quartile of PROP taste score for PA/PA homozygotes (tasters).

Homozygote taster (PA/PA)	Manual social class (n = 646)	Antisocial activities (n = 721)	Depressed tendency (n = 710)
1	69	29	38
	(42.07)	(15.85)	(21.47)
2	65	31	32
	(36.93)	(15.82)	(16.58)
3	71	23	40
	(46.10)	(13.37)	(23.39)
4	71	32	53
	(46.71)	(18.82)	(31.36)
linear test, p	0.2	0.6	0.6

Note, reference groups are not shown. 1, 2, 3, 4 represent grouping by quartiles of PROP taste test (general labelled magnitude scale).

## Discussion and conclusion

As a study containing data on n = 4178 PROP taste test scores and concurrent *TAS2R38 *haplotypes, analysis in the ALSPAC cohort is the largest study to date to address the question of the haplotypic association with tasting ability. This is the first instance of the application of the gVAS to children in this manner and has shown this to be a consistent approach to the measurement of tasting ability. Resolution of common haplotypes across the *TAS2R38 *locus revealed variants adhering to Hardy Weinberg Equilibrium and for which frequencies were close to those reported elsewhere [12,21].

Analysis of the PROP taste test scores in the ALSPAC cohort has replicated previous findings of association between variation at the *TAS2R38 *locus and response bitter taste test challenge. As predicted by the findings of Kim et al [12], the common PA haplotype predicted those able to taste the bitter compound PROP when at least one copy was carried. Alternatively, homozygotes of the other common haplotype, AV, (haplotypes AV and PA accounting for >90% of 2N) exhibited clear patterns of taste insensitivity to the bitter PROP compound.

Importantly, the predicted intermediate tasting ability of those carrying the rare AA haplotype [17] (in cases excluding heterozygotes with one copy of the common and "taster" defining PA haplotype) appears to be demonstrated in the ALSPAC cohort.

As yet unreported, we were also interested in the presence and patterning of variation in taste test scores within the genetically prescribed tasting groups for PTC/PROP. We performed an investigation into the distribution of PROP taste test scores within genetically homogeneous groups of tasters (homozygote PA individuals) and non-tasters (homozygote AV individuals). This revealed that three select factors (depression, social class and antisocial behaviour) thought to influence the response of children to bitter taste challenge did not appear to be associated with the variation in taste test score in these groups.

On the basis of current observations, it is suggested that physiological differentiation, likely derived from further genetic factors [8,9,11,22], may form the basis of variation in taste score within groups previously defined as "tasters/nontasters" by *TAS2R38 *haplotypes. These further possible physiological factors include not only alternative taste receptor complexes, but possibly factors previously associated with the existence of "super tasting" ability [23-26]. Importantly, this notes that (i) residual variation in this particular taste test score, after previously recognised haplotype groupings, is likely to be due to other physiological mechanisms and (ii) that there may be a degree of phenotypic continuity in this trait recognised classically as bimodal in character. In addition to this, we observed that in the overall population, the classification of all individuals into the broad categories "taster/nontaster" may not be appropriate. Evidence from this study suggests that the carriage of particular haplotypic combinations is associated with intermediate bitter compound tasting ability.

## Methods

The Avon Longitudinal Study of Parents and Children (ALSPAC) is a geographically based cohort that recruited pregnant women residing in Avon with an expected delivery date between 1^st ^April 1991 and 31^st ^December 1992. 14 541 pregnant women were initially enrolled, with 14 062 children born. This represents 80–90% of the eligible population [27,28]. Of these children, 13 988 were alive at 12 months. From this, two sampling frames were to be adhered to (i) the maximal population sample for haplotypic reconstruction (including children with DNA samples and mothers with DNA samples where a child's DNA sample was missing) and (ii) the analysis data set including just children with at least one genotype and concurrent PROP taste test results. This formed a maximal baseline population of n = 12371 (reduced to n = 12234 with the loss of one sib from twin pairs) for which, in order to allow investigation, ethical approval was obtained from the ALSPAC Law and Ethics Committee, and local research ethics committees.

### PROP test

Paper disks impregnated with PROP have been shown to be a crude but rapid way to test responses to PROP in large groups [19]. As part of a face-to-face clinic session held when the children were aged 10 years, a nutritionist interviewed them about their diet. Following this, the nutritionist proceeded to assess the subject's reaction to a bitter (PROP) challenge using a general visual analogue scale (gVAS) [29].

The disks were prepared by soaking circular pieces of filter paper (Whatman #1) in a saturated solution of PROP (at near boiling temperature) and then drying them. The PROP crystallizes into the paper thus allowing the paper to serve as a convenient way to permit a subject to taste a limited quantify of PROP crystals. The PROP crystals go into solution in the subject's saliva and produce a high concentration of PROP at the taste receptor sites. The paper produces bitterness approximately equivalent to a solution of .0032 M, close to the highest concentration of PROP that will remain in solution when PROP solutions are refrigerated for storage. The purpose of using a high concentration for screening is that PROP functions for nontasters, medium tasters and supertasters diverge; thus the highest practical concentration of PROP produces the most accurate sorting.

In this test, the nutritionist explained to the child that they were going to taste a piece of paper and would then mark how strong they thought the sensation of the taste was on a line. The child was asked to name their senses and was prompted if he/she could not. The nutritionist then asked the child to describe the loudest, most intense sound they had ever heard and the brightest, most intense light they had ever seen. They then pointed to the scale on the datasheet (a 10 cm line), explaining that the left end of the scale represented no sensation and the right end represented the most intense sensation, explaining again the most intense sensation as the loudest sound or brightest light that they had just described. The child was then asked to point on the line where a whisper and where a shout would go on the scale. If children understood the instructions, a whisper would be rated as less intense than a shout which, in turn, would be rated less intense that the loudest sound. Once children rated the sounds in the correct order, they were asked to place a disc of PROP paper on their tongue and move it around for about 10 seconds. The child was then given a pen and asked to make a mark on the line. Once the child had left the room the nutritionist measured how far from the left the child had marked (10 cm being the maximum).

Of note, the instructions regarding the whisper were used to check that the child understood how the scale worked and which end represented the largest magnitude of stimulation in regard to the senses.

### PROP taste test consistency

Within the focus at 10 years of age follow-up screens, a small subset of the ALSPAC cohort who under took taste tests were re-invited to perform the test again (i.e. a repeat of the clinic at 10 years). Of 237 children assigned re-invitation (approximately 3% of the total sample set) 203 children completed the test the first time and 196 the second time. In total, 168 children performed the test twice. These provided data for the assessment of internal consistency for the PROP taste test. Consistency was assessed by analysis of the correlation between test scores at both measurements and by the regression of repeat test data.

### Genotyping

Genetic variants were selected on the basis of previous literature indicating their association with PTC taste test scores. SNPs to be investigated here are the A49P and V262A variants of the *TAS2R38 *gene on Ch7 (rs713598 and rs1726866 respectively). Both of these are non-synonymous protein change causing variants the former leading to a Proline/Alanine change at amino acid position 145, whilst the latter leads to a Alanine/Valine change at amino acid position 785. As such, these are taken as likely to be functionally significant in relation to the action of the *TAS2R38 *gene (although the actual implication of these variants are unknown) [12].

DNA, from cord blood or peripheral blood was extracted and processed as described previously [30]. SNPs were genotyped using the KASPar chemistry which is a competitive allele specific PCR SNP genotyping system using FRET quencher cassette oligos[31]. All genotyping was performed by KBioscience [32].

### Psychosocial factors

Social class was derived using OCPS classifications [33] with information taken from a self completed questionnaire administered to the mothers at 32 weeks of pregnancy. This information yielded a 6-level variable I, II, III non-manual, III manual IV, V, which was dichotomised centrally for a manual/non-manual split.

For the assessment of depression the children were given a series of envelopes with statements written on them about how they might have been feeling or acting in the previous two weeks. This was done at the same clinic session. Depression was assigned in cases where 6 or more indicators of depression existed. This represented the top quartile of the population distribution. The statements used have been taken from the Short Mood and Feelings Questionnaire [34], which has been designed to provide a rapidly administered questionnaire for use in epidemiological studies.

Lastly, antisocial behaviour was defined by the presence of any 1 of 11 antisocial activities report by the child during the same clinic session, covering a range of behaviours including stealing, cruelty to animals, smoking, drinking, taking drugs and a series of dummy questions. Questions were derived from the self-reported antisocial behaviour for young children questionnaire [35]. A variable was then derived indicating whether any such activity had been carried out.

For the purpose of analysis of the distribution of taste test scores by social class, antisocial behaviour and depression, tasting scores were organised into quartiles.

### Statistical analysis

Table 1. summarises haplotypes and predicted taster status identified in relation to previous literature (i.e. an individual's status as either a 'taster' or 'non-taster' of bitter compounds). Haplotype names 'PAV' and 'AVI' refer to recognised 'taster' and 'non-taster' states respectively [12] and are specifically derived from their protein coding sequences. In the context of this study, haplotypes 'AVI' and 'PAV' have been renamed AV and PA respectively. An individual was therefore designated a 'non-taster' if they carried two copies of AV and a 'taster' if they carried at least one copy of PA.

**Table 1:** Prediction of tasting ability by *TAS2R38 *haplotype

Kim et al haplotype	Present Study Label	P49A rs713598	A262V rs1726866	[V296I]	Taster Status
AV [I]	AV	G	T	[A]	Non-taster if homozygote
AA [V]	AA	G	C	[G]	Small effects on non-taster status when heterozygote AVI/AAV
PV [I]	PV	C	T	[A]	unknown
PA [V]	PA	C	C	[G]	Taster if homozygote/heterozygote

(Haplotypes taken from Kim et al [8]. Haplotype labels are derived from amino acid coding patterns. Square brackets indicate exclusion of variant in present study due to linkage disequilibrium with included variants.)

Previous results [12] demonstrated the existence of the variant site *TAS2R38 *V296I (34 bases from *TAS2R38 *V262A) in total linkage disequilibrium (LD) with *TAS2R38 *V262A in a European population. In light of this, it was felt appropriate that the genotyping of *TAS2R38 *P49A and *TAS2R38 *A262V alone would allow the effective tagging of common haplotypes in this region.

Having collected genotype data, haplotypes were constructed using the programme PHASE (version 2.02, [36], ). This software employs a Bayesian method for the reconstruction of chromosomal phase using genotype data and generates counts and frequencies of observed haplotypes. The underlying method in this approach is a Markov Chain-Monte Carlo procedure in which the probability of preceding observations (in this case unambiguous phase information) allows population genetic inference about unresolved haplotypic phase. Having run phase, posterior probabilities of phase reconstruction were employed to allow the incorporation of a haplotype accuracy cut off for further analysis. For the purpose of taste test analysis, this was constrained at 80% accuracy for the reconstruction of PTC haplotypes.

Initial analysis considered median (due to skewed distribution of PROP taste test scores) PROP taste test scores by the haplotypic combination (diplotype) carried by those in the ALSPAC sample. Based on Kim and colleagues [12], our prior hypothesis was that bitter tasting ability would differ according to taster status as defined by haplotypic complement (homozygous AV = non-taster, heterozygous or homozygous PA = taster, other = excluded). This model formed the basis of test groups in taste test scores were included for analysis. To assess the distribution of PROP taste test results for the rarer AA haplotype, a further grouping was generated as defined by any carriage of the AA haplotype (excluding the common and taster predicting PA haplotype) versus the rest of the sample. This grouping was largely designed on the basis of the findings of Bufe et al [17] and was deigned to assess the potentially intermediate tasting ability of those carrying the rare AA haplotype.

Due to the expected bimodal distribution of PROP taste test results, the non-parametric rank sum approach to testing for differences between the properties of test groups was used in this analysis. This also prompted the use of robust standard errors in the presentation of mean pairwise differences in taste test score by diplotypes along with their 95% CI. Test statistics for the analysis of quartiles of PROP taste test with social class, depression and antisocial behaviour were generated by via simple trend analysis.

All analyses were performed in STATA version 8.2.9 and SPSS version 9.12.

## Competing interests

The author(s) declare that they have no competing interests.

## Authors' contributions

NT performed the statistical analysis and coordinated the writing of the paper and acts as guarantor

JH sourcing and piloting of materials and assisted in project management of data collection

INMD inputs to improvements phase of analyses and manuscript

SR production of DNA bank and overseeing provision of DNA and linking genetic data

LB was involved in original data collection and contributed to paper revision

PE – lead the team that administered the PROP challenge having taken advice from LMB on the correct procedure, she was fully involved in the writing of the manuscript.

GDS analysis plan and revising manuscript
